# The “POP-IT” ear deformity: early recognition and non-surgical management of a novel neonatal auricular deformation

**DOI:** 10.1007/s00405-026-10503-6

**Published:** 2026-08-03

**Authors:** Daniel Hilewitz, Binyamin Shachor, Sagit Meshulam, Asaf Olshinaka, Dean Ad-El, Dafna Shilo Yaacobi

**Affiliations:** 1 Gray Faculty of Medical and Health Sciences, Tel Aviv University, Tel Aviv, Israel; 2 Department of Plastic Surgery and Burns, Rabin Medical Center, Petah Tikva, Israel; 3 Pediatric Plastic Surgery and Burns Unit, Schneider Children’s Medical Center, Petah Tikva, Israel

## Abstract

**Background:**

Congenital auricular deformities are common in newborns, yet many remain underdiagnosed despite the availability of effective early non-surgical correction. We describe a previously unreported auricular deformation, termed the “POP-IT” ear deformity, and present our early clinical experience with non-invasive management

**Methods:**

A retrospective case series of neonates diagnosed with the POP-IT ear deformity and treated with auricular molding between 2024 and 2025 was conducted.Demographic data, deformity characteristics, treatment timing, duration, outcomes, and follow-up were analyzed.

**Results:**

Seven neonates (6 males, 1 female) were included. The mean age at treatment initiation was 12.0 ± 0.3 days. The deformity was unilateral in six patientsand bilaterally asymmetrical in one. Five patients had associated auricular deformities. The mean gestational age was 38.6 ± 0.2 weeks . Desired correction was achievedwithin a mean of 7.0 ± 0.3 days, followed by continued molding for maintenance, with a total treatment duration averaging 4.0 ± 0.3 weeks. No complications were observed.Mean follow-up was 18.5 ± 5 months, with stable aesthetic outcomes in all patients

**Conclusions:**

The POP-IT ear deformity represents a distinct neonatal auricular deformation that can be effectively corrected using early non-surgical molding. Prompt recognition and intervention lead to rapid correction and durable outcomes, potentially obviating the need for future surgical treatment.

## Highlights

POP-IT deformity is a previously undescribed neonatal auricular deformation.

Early non-surgical molding achieved correction within a mean of 7 days.

No skin complications or relapse were observed during follow-up.

Most patients had associated auricular anomalies, supporting comprehensive neonatal ear assessment.

Early recognition and molding may prevent future surgical correction.

## Introduction

Congenital auricular anomalies affect up to 30% of newborns; however, only a minority resolve spontaneously, and many remain undiagnosed due to limited awareness among parents and healthcare providers and the perception that such anomalies are primarily cosmetic [1, 2]. Early identification is therefore critical, as the neonatal period provides a unique window during which auricular cartilage is highly malleable because of elevated maternal estrogen and hyaluronic acid levels, enabling effective non-surgical correction [3, 4].

Auricular anomalies are broadly classified into malformations, which involve structural deficiencies requiring surgical reconstruction, and deformations, in which fully developed ears exhibit abnormal shape and can often be corrected with molding techniques [2, 5]. Early non-surgical ear molding has emerged as a safe and effective approach for managing auricular deformations, achieving high parental satisfaction and potentially avoiding later operative intervention [1, 6, 7].

**Fig. 1:**
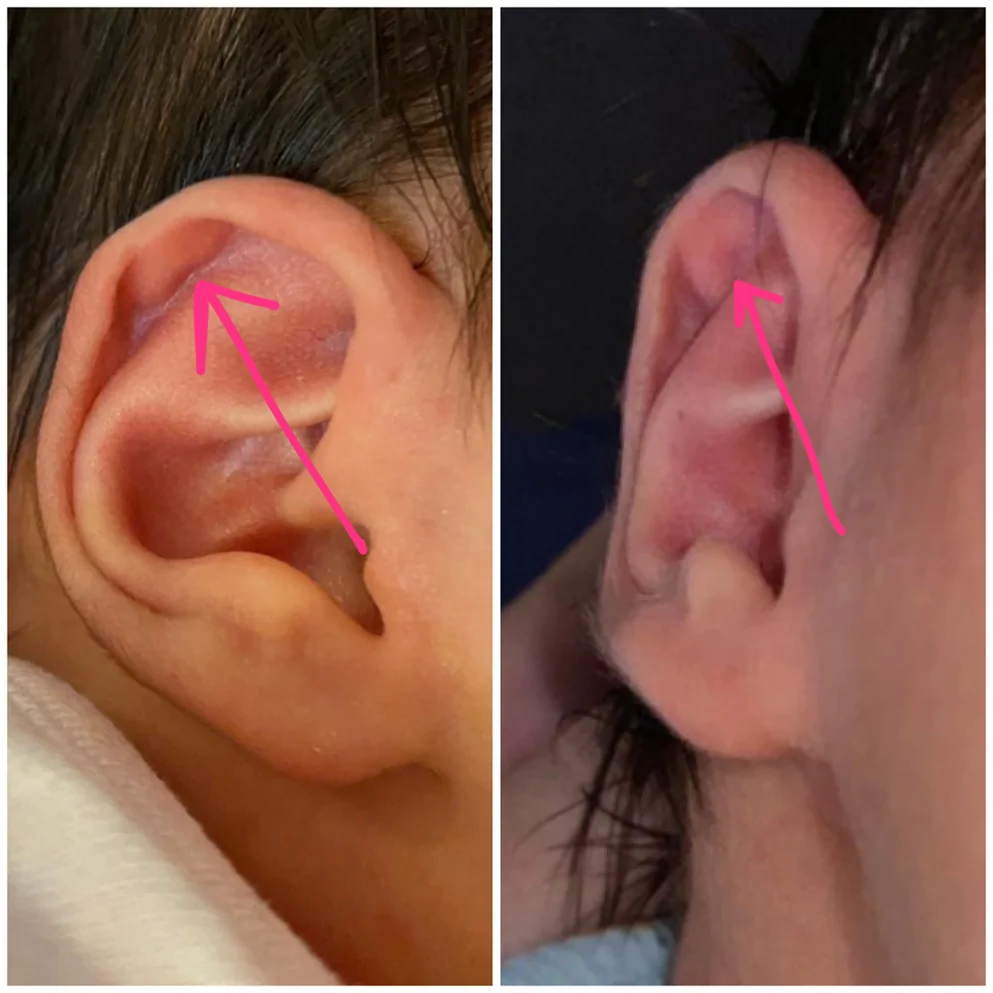
Annotated schematic illustration of the POP-IT deformity

While numerous deformational variants have been described, including lop ear, Stahl ear, prominent ear, cryptotia, concave concha [8], and helical rim anomalies, we introduce a previously unreported phenotype termed the “POP-IT” ear deformity, named for its resemblance to the popular sensory fidget toy [1, 5, 9]. This deformity primarily involves abnormal contouring of the scapha and helix and may be mistaken for a helical rim deformity, although its anatomical configuration is distinct [5]. Specifically, the deformity is characterized by an outward convexity of the scaphal region accompanied by abnormal helical folding, producing a contour reminiscent of a “popped” segment of a sensory fidget toy. Unlike classic helical rim deformities, the primary abnormality is centered within the scapha–helix complex while preserving the remaining auricular architecture (Fig. 1).

The aim of this study is to describe the clinical characteristics of the POP-IT ear deformity and to report our experience with early non-invasive treatment using auricular molding in a series of neonates.

## Materials and methods

We conducted a retrospective case series of neonates diagnosed with the POP-IT ear deformity and treated between January 2024 and December 2025 at a pediatric plastic surgery clinic. Inclusion criteria were: (1) diagnosis of POP-IT ear deformity within the neonatal period, (2) initiation of non-surgical molding therapy, and (3) available follow-up of at least four months post treatment. Neonates with auricular malformations or syndromic diagnoses were excluded.

Demographic and clinical variables included gender, gestational age, age at treatment initiation, laterality, presence of associated auricular deformities, general health status, duration of treatment, time to initial correction, and follow-up duration. Continuous variables are presented as mean ± standard deviation (SD).

Auricular molding was initiated as early as possible after diagnosis using retractor-based molding of the scapha and helix, stabilized with adhesive taping to the scalp. The molding construct by the EarWell System (Becon Medical, Naperville, IL), each was individually tailored to each patient using retractors positioned to recreate normal scaphal concavity and helical contour. Adhesive fixation to the scalp maintained constant corrective forces throughout treatment, with weekly adjustments performed according to the achieved correction. The aim was to recreate normal scaphal concavity and helical contour. Parents were instructed regarding daily care and monitoring. Weekly clinical evaluations were performed.

Once the desired contour was achieved, molding was continued for maintenance to stabilize cartilage remodeling; therefore, total treatment duration exceeded the time required to reach initial correction.

The primary outcome was achievement of satisfactory auricular contour as judged clinically by the treating surgeon and parental satisfaction. Secondary outcomes included time to correction, total treatment duration, complications, and stability at follow-up.

The study was approved by the institutional review board, and informed parental consent was obtained for treatment and data collection.

## Results

Seven neonates were included (6 males, 1 female). The mean age at initiation of treatment was 12.0 ± 0.3 days. The mean gestational age was 38.6 ± 0.2 weeks. Deformity was unilateral in six cases (86%) and bilaterally asymmetrical in one (14%). Five neonates (71%) had associated auricular deformities, while in two cases (29%) the POP-IT deformity was isolated. All infants were otherwise healthy, with no reported skin sensitivity or underlying medical conditions.

Initial satisfactory correction was achieved within a mean of 7.0 ± 0.3 days from treatment onset. Molding was then continued for maintenance, with total treatment duration averaging 4.0 ± 0.3 weeks. No skin ulceration, dermatitis, or other complications were observed.

Follow-up ranged from 4 months to 24 months, with a mean of 18.5 ± 5 months. All patients demonstrated stable correction without relapse.

## Discussion

**Fig. 2:**
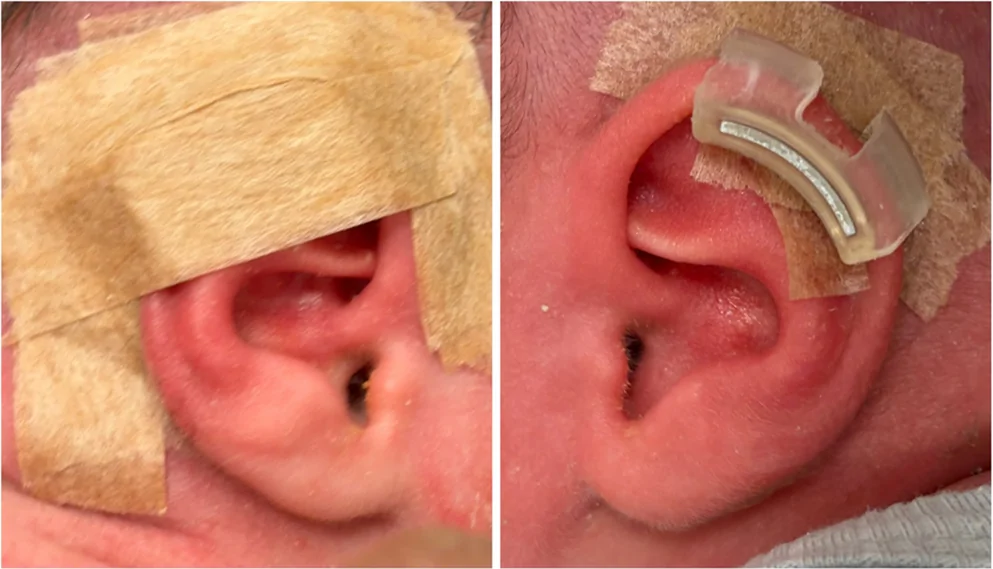
The molding construct in place, demonstrating retractor positioning and adhesive fixation during treatment

**Fig. 3:**
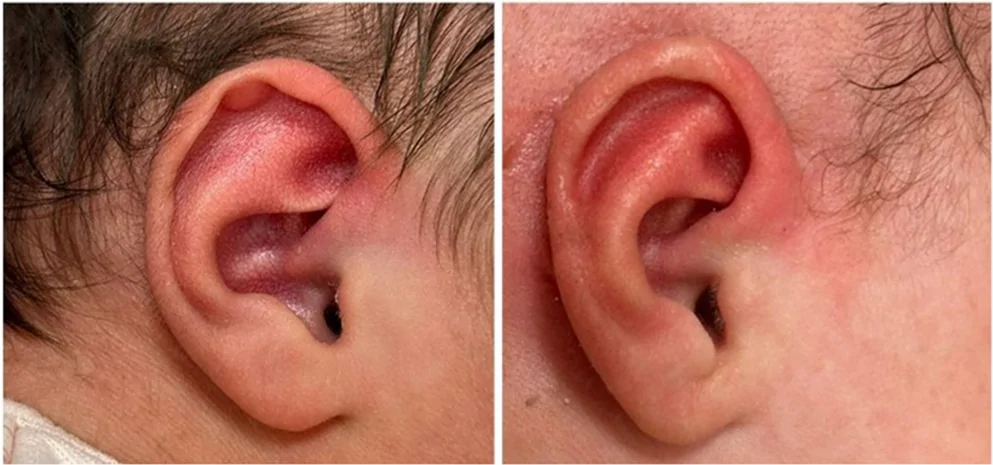
Pre- and post-treatment (1 month) photographs of POP-IT deformity

**Fig. 4:**
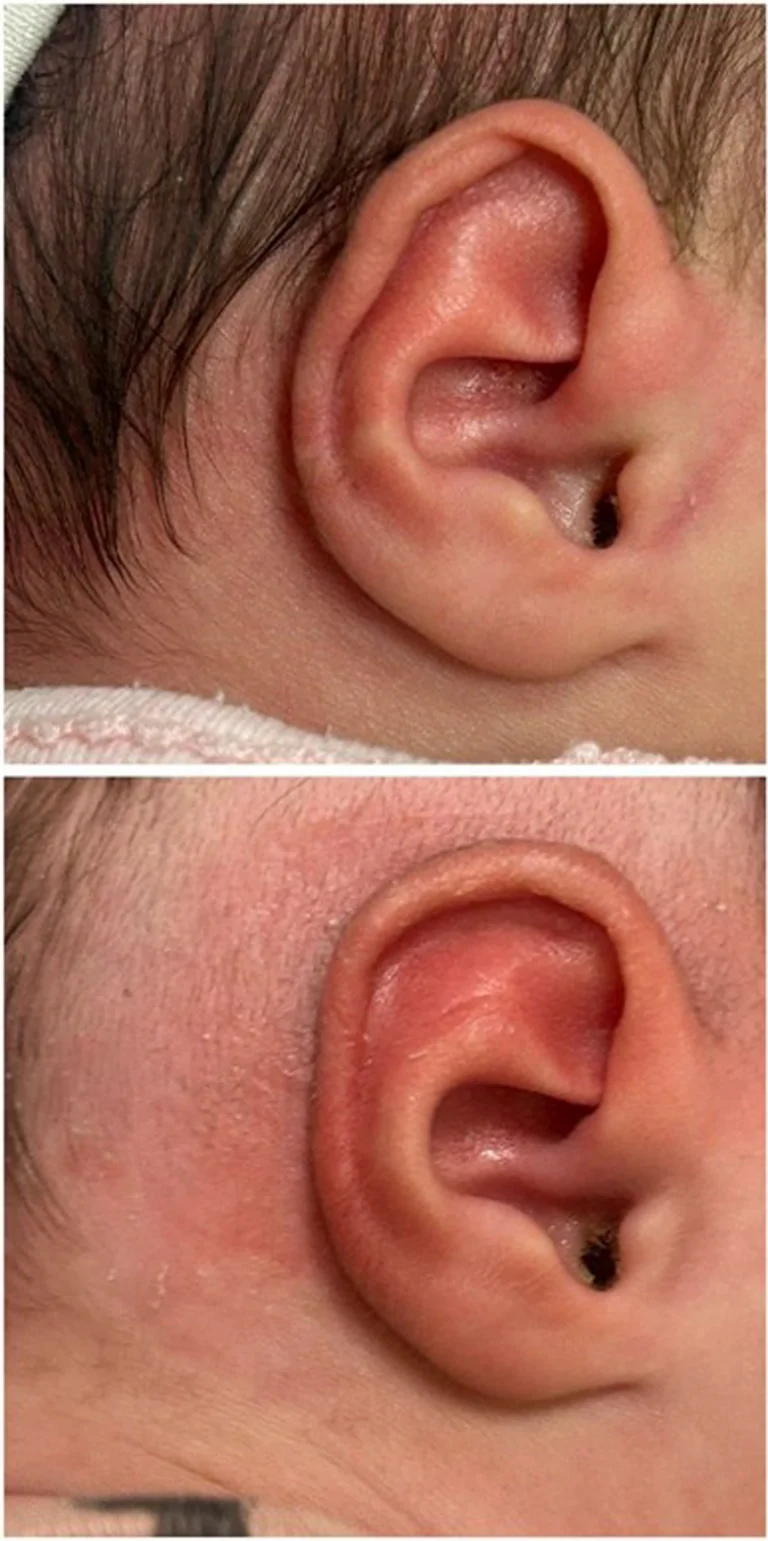
Pre- and post-treatment (2 months) photographs of POP-IT deformity

**Fig. 5:**
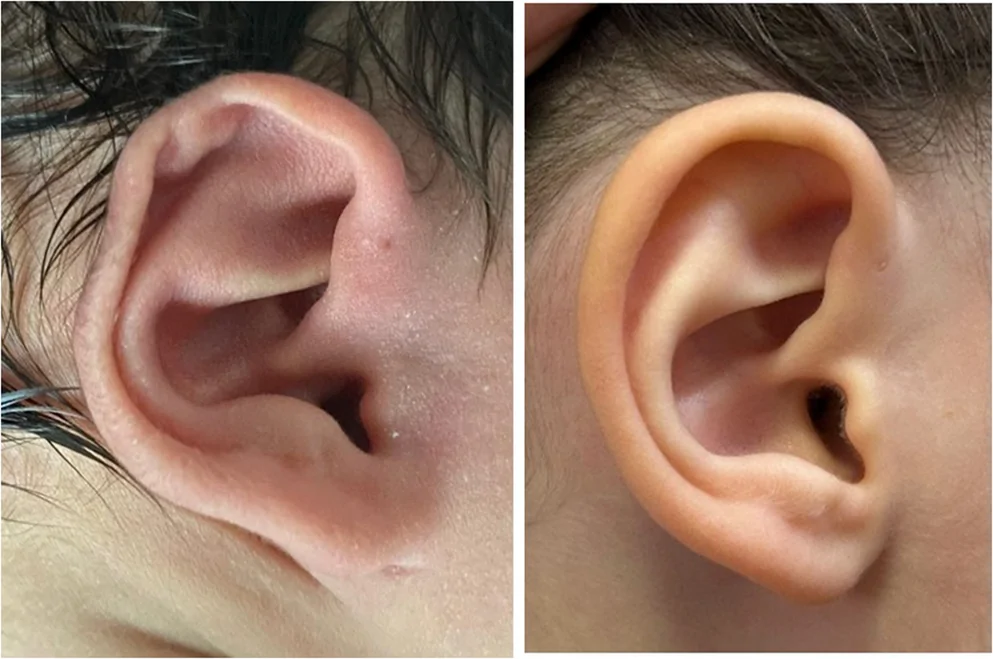
Pre- and post-treatment (3 years) photographs of POP-IT deformity

This study introduces the POP-IT ear deformity as a distinct auricular deformation phenotype and demonstrates that it can be effectively corrected through early non-surgical molding. In our cohort, rapid correction was achieved within approximately one week, with durable outcomes maintained during long-term follow-up (Figs. 2, 3, 4 and 5).

The high prevalence of associated deformities in our series suggests that the POP-IT deformity may coexist with other auricular shape anomalies, reinforcing the importance of comprehensive auricular assessment in neonates [2]. Like other deformations, its responsiveness to molding highlights the plasticity of neonatal cartilage and the critical importance of early referrals [3, 4]. 

Our findings align with prior literature demonstrating that early initiation of ear molding leads to shorter treatment duration and higher success rates [10]. Notably, despite a mean initiation age of 12 days, slightly beyond the immediate postnatal period of the first few days after birth, excellent outcomes were still achieved, suggesting a potentially wider therapeutic window for this deformity [1, 10]. 

Compared with surgical otoplasty, non-surgical molding offers clear advantages: absence of anesthesia, no scarring, minimal risk, low cost, and immediate psychosocial benefit for families. Early correction may eliminate the need for later operative intervention and its associated morbidity. In addition, early molding can correct subtle contour abnormalities and scaphal–helical deformities thin are often challenging to address surgically, further underscoring its unique therapeutic value.

The absence of complications in our cohort supports the safety of this approach when performed under close supervision. The durability of correction over an average follow-up exceeding 18 months further underscores its effectiveness.

Limitations include the small sample size and retrospective design, inherent to the rarity and novelty of the deformity. Larger studies are needed to better define incidence, anatomical variability, and long-term outcomes. Objective aesthetic scoring systems and quality-of-life measures would further strengthen future evaluations.

Nevertheless, this initial series provides important clinical insight and establishes a framework for recognition and management of this newly described auricular deformation.

## Conclusions

The POP-IT ear deformity represents a novel neonatal auricular deformation involving abnormal scaphal and helical contouring. When recognized early, it can be safely and effectively treated using non-invasive auricular molding, achieving rapid correction and durable aesthetic results. Increased awareness among neonatologists, pediatricians, and plastic surgeons is essential to enable timely referral and optimize outcomes, potentially obviating the need for future surgical correction.

## Data Availability

The data that support the findings of this study are available from the corresponding author upon reasonable request.
